# Successful surgical management of post-penetrating or deep lamellar keratoplasty Acquired Corneal Sub-Epithelial Hypertrophy (ACSH): A case series

**DOI:** 10.1016/j.ijscr.2020.01.054

**Published:** 2020-02-06

**Authors:** Abdulmohsen Almulhim, Moustafa S. Magliyah, Abdullah Alfawaz, Jose Manuel Vargas, Abdulrahman Al-Muammar, Hind Alkatan

**Affiliations:** a Ophthalmology Department, College of Medicine, Al-Jouf University, Sakakah, Al-Jouf, Saudi Arabia; b Ophthalmology Department, Prince Mohammed Medical City, Al-Jouf, Saudi Arabia; c Ophthalmology Department, College of Medicine, King Saud University, Riyadh, Saudi Arabia; d Cornea and Anterior Segment Division, King Khaled Eye Specialist Hospital, Riyadh, Saudi Arabia; e Pathology Department, College of Medicine, King Saud University, Riyadh, Saudi Arabia

**Keywords:** Acquired Corneal Sub-Epithelial Hypertrophy, Corneal opacity, Salzmann’s nodular degeneration, Penetrating keratoplasty, Lamellar keratoplasty, Superficial peeling, Superficial keratectomy, Case series

## Abstract

**Introduction:**

Acquired Corneal Sub-Epithelial Hypertrophy (ACSH) has been described in patients with peripheral superficial corneal opacities following penetrating keratoplasty and might present similar to Salzmann’s nodular degeneration (SND) or peripheral hypertrophic sub-epithelial corneal degeneration (PHSCD). We describe the clinical presentation, topographic findings and the surgical outcome of three cases, which fit the appearance and characteristics of ACSH.

**Presentation of cases:**

Three patients (3 eyes) with paracentral or peripheral corneal opacification were reviewed to describe their clinical examination (SL), morphology of the opacity (depth, diameter and density) and document their topographic changes before and after surgical intervention by peeling of the epithelium with or without superficial keratectomy under the microscope in addition to brief description of their histopathological examination.

**Discussion:**

All 3 cases were secondary to corneal procedures [Penetrating keratoplasty (PKP) in 1 for pseudophakic bullous keratopathy and deep anterior lamellar keratoplasty (DLK) in 2 for advanced keratoconus]. All cases presented with reduced vision, astigmatic changes in topography or manifest refraction. The visual acuity, symptoms, and topographical findings all improved after treatment. Histopathologically, all cases fit the newly described entity of ACSH.

**Conclusion:**

Careful clinical judgement guided by corneal topography are needed for proper the diagnosis of acquired corneal opacification that results in reduction of vision to identify ACSH from other similar conditions (PHSCD and SND). Peeling of the thickened epithelial and sub-epithelial tissue is curative avoiding the need for corneal re-grafting.

## Highlights

• The differential diagnosis of superficial corneal opacities is challenging.
• It includes 3 described overlapping entities: SND, PHSCD, and Acquired Corneal Sub-Epithelial Hypertrophy (ACSH).
• Our case series match the newly proposed entity of ACSH.
• ACSH should be accurately identified following keratoplasty to be successfully managed by simple surgical peeling without corneal re-grafting.

## 1 Introduction

Peripheral hypertrophic sub-epithelial corneal degeneration (PHSCD) is an idiopathic condition characterized by the presence of fibrotic changes in the sub-epithelial corneal layer which leads to corneal flattening and astigmatism. It is typically bilateral, symmetrical, peripheral, and sub-epithelial in location [1,2]. This entity is also similar to Salzman Nodular Degeneration (SND) [3,4]. Recently Al-Rajhi et al. described an entity of an acquired corneal opacification that share few similarities with PHSCD -and proposed the term Acquired Corneal Sub-epithelial Hypertrophy (ACSH)-, but with differences from SND and PHSCD in age, etiology (being mostly acquired), and density in addition to their peculiar arcuate shape in most of the cases [4]. The research work has been reported in line with the PROCESS criteria for a case series [5].

## 2 Methods

This case series was prepared in accordance with the ethical standards of the human ethics in accordance with the Helsinki Declaration. No IRB approval is needed for case reports. However, a general informed consent was taken from all patients, which includes permission for anonymous reporting. The authors have no financial disclosures to declare nor conflict of interest in relation to this case series. No financial funds involved. Research registry of the case series: #researchregistry5315. We retrospectively report three cases with identical clinical and histopathological features to their described entity, who were similarly treated with superficial corneal peeling in 2 centers: King Abdulaziz University hospital at King Saud University (two cases) and King Khaled Eye Specialist Hospital (one case). Both centers are government academic tertiary eye care hospitals. The surgical procedure was performed under topical anaesthesia in each corresponding center where the patient originally presented by the primary caring consultant ophthalmologist, all of whom had long experience in the field of cornea and external disease and are included as co-authors. The obtained specimens were histologically examined by the senior author for confirmation of the specific entity of ACSH. The follow-up periods for the patients have been variable according to their initial presentation date however, they all share a common satisfactory post-operative result. No long-term follow-up was necessary in these cases since a reasonable clarity of the cornea was shortly obtained after the surgical intervention.

## 3 Results

3 consecutive cases have been included with the following description of each case.

### 3.1 Case 1

**Fig. 1:**
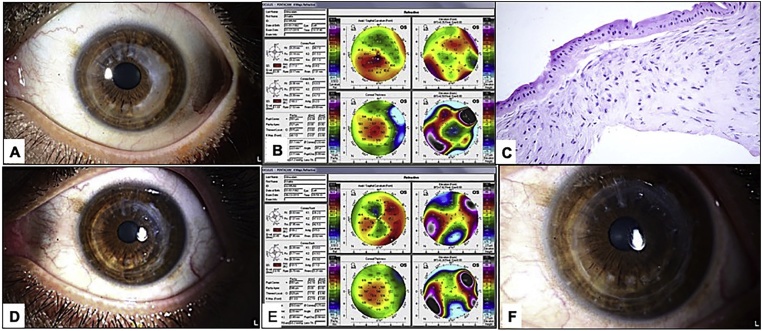
A: Clinical photo of the left eye showing sutur-less graft with clear visual axis with arcuate-shaped avascular opacity of moderate density mostly confined to the temporal aspect graft extending from 12 to 5 o’clock in otherwise quiet eye. B: Topography of the same eye showed an increase of the corneal thickness on the pachymetry map (corresponding to the elevated opacified white lesion), on the sagittal curvature map there is marked flattening in the involved area giving irregular surface, and variable areas of elevation in the front elevation map but no abnormality seen on the back elevation over the area of the opacity. C: Histopathology photo of the excised tissue showing the corneal epithelium with absent Bowman’s layer and sub-epithelial fibrous tissue (Original magnification ×200 Hematoxylin and eosin). D: Clinical photo of the same eye 1 week after superficial peeling showing a clear graft with significant disappearance of the temporal corneal opacity and smooth surface. E: Left eye topography 2 weeks after the peeling showing better symmetry of the curvature map and regularization of both anterior elevation and thickness. F: The same cornea has maintamined clarity with no recurrance of tha opacity 3 months after the procedure.

A 36-year-old gentleman who is known to have advanced keratoconus for which deep anterior lamellar keratoplasty was done in his left eye for visual rehabilitation. After removal of all sutures and satisfactory period of several years, during which his uncorrected vision (UDVA) was measuring 20/30 in his left eye, the patient started to notice a progressive blurring of vision. At presentation, his left eye UDVA was 20/80 improving with pinhole to 20/40. The intraocular pressure (IOP) was 13 mmHg. The conjunctiva was quite but the cornea was showing an arcuate opacity around the suture-less edge of the graft temporally resembling a corneal keloid like picture with a clear center (Fig. 1a). The right was stable with central faint corneal scar and visual acuity that measures 20/28.5. Topography of the same eye showed an increase of the corneal thickness corresponding to the elevated opacified white lesion, irregular corneal surface on the sagittal curvature map, and variable areas of elevation in the front elevation map but no abnormality seen on the back elevation (Fig. 1b).

The surgical intervention was performed by simple gentle manual peeling of the opacified thickened epithelium and sub-epithelial tissue aiming at achieving a clear and smooth stromal plane without causing any surface irregularity followed by application of Mitomycin C (MMC) 0.02% for 2 min with copious irrigation of the corneal surface with Balanced Saline Solution (BSS). After the procedure bandage contact lens was applied to allow corneal epithelial defect to heal and to ease the post-operative recovery for the patient under the coverage of prophylactic broad-spectrum antibiotic drops as well as steroid drops to prevent the theoretical risk of recurrence from the robust wound healing process. The excised tissue showed variably thickened corneal epithelium with no Bowman’s layer and sub-epithelial fibrous tissue (Fig. 1c). There was no inflammation or evidence of neovascularization.

One week following the procedure, the patient showed an improved measured visual acuity I the left eye: (UDVA: 20/40). The epithelial defect healed with significant disappearance of the corneal opacity and better graft clarity (Fig. 1d). Repeated left eye topography 1 week later (after BCL removal) showed significant improvement and better symmetry of the curvature map and regularization of both anterior elevation and thickness maps when compared with pre-operative topography (Fig. 1e). The left cornea remained clear 3 months after the procedure in his most recent follow up visit (Fig. 1f).

### 3.2 Case 2

**Fig. 2:**
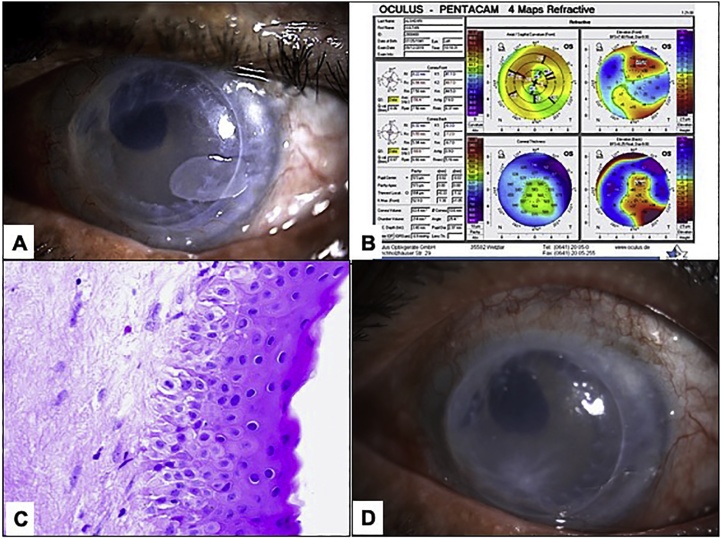
A: Clinical photo of the left eye showing suture-less minimally edematous graft with avascular temporal hypertrophic superficial corneal lesion of mild density straddling the graft-host junction. B: Topography of the same eye showing increased corneal thickness over the area of the lesion and irregular corneal surface. C: Histopathological photo of the peeled thickened corneal epithelium, absent Bowman’s layer and identical sub-epithelial hypocellular fibrous tissue (Original magnification ×400 Hematoxylin and eosin). D: Postoperative clinical photo taken 3 weeks after the procedure with no evidence of the temporal opacity with more corneal edema for future re-grafting.

A 78-year-old male was referred to the cornea service at King Khaled Eye Specialist Hospital four years prior to this recent presentation as a case of Pseudophakic Bullous Keratopathy (PBK) in the left eye with Best corrected visual acuity (BCVA) measuring 20/300. He had Penetrating keratoplasty (PKP) and his BCVA improved to 20/60. Four years later, his BCVA dropped to 20/200 and his left eye slit lamp examination (Fig. 2a) showed a temporal hypertrophic superficial corneal lesion corresponding to an increased corneal thickness and irregular corneal surface on corneal topography (Fig. 2b). Over a period of one year the cornea started to show signs of impending failure in form of corneal edema with no signs of rejection so as a preparatory step before going for endothelial keratoplasty the surgeon elected to do simple peeling of this localized opacified thickened epithelium and sub-epithelial tissue. The excised corneal opacified lesion showed remarkably thickened epithelium, absent Bowman’s layer, and hypocellular fibrous tissue in the sub-epithelial area (Fig. 2c). His BCVA improved to 20/100 with no evidence of recurrence of the opacified area but the cornea was more edematous due to endothelial failure necessitating endothelial keratoplasty in the future for visual rehabilitation (Fig. 2d).

### 3.3 Case 3

**Fig. 3:**
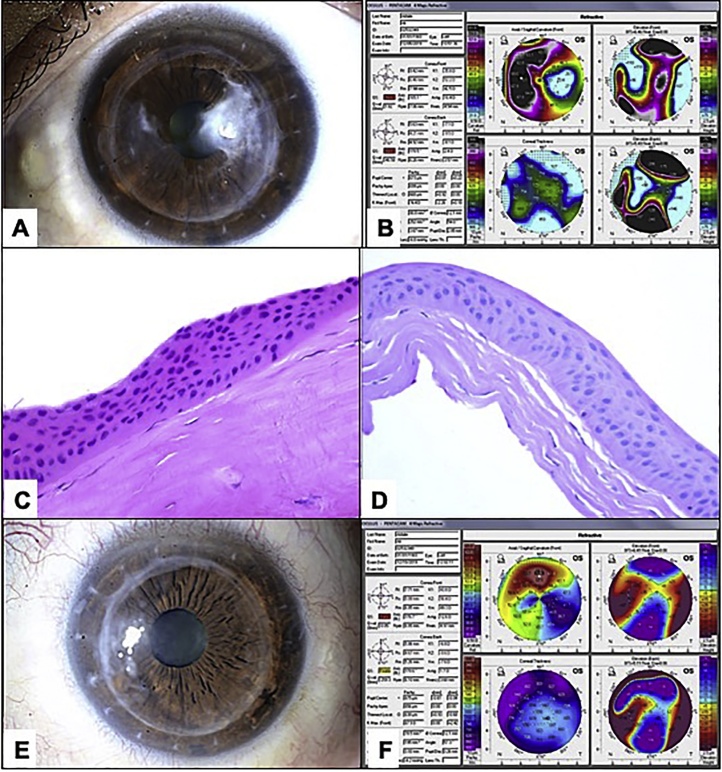
A: Clinical photo of the left eye showing suture-less compact corneal graft with none vascularized elevated corneal opacity more prominent temporally over the graft extending to the visual axis. Minimal opacification is also seen nasally. B: Topography of the same eye significant flattening over the area of corneal opacity temporally with total anterior astigmatism of 16 diopters, with anterior elevation corresponding to the area of opacity. On pachymetry there was marked thickening at the area of the opacity temporally. C: Histopathological photo of the peeled thickened corneal epithelium with sub-epithelial fibrosis (Original magnification ×400 Hematoxylin and eosin). D: The same peeled superficial corneal tissue with clearly absent Bowman’s layer (Original magnification ×400 Periodic acid Schiff). E: Clinical photo taken 3 weeks after the procedure showing clear graft with no visible opacity and clear visual axis. F: Topography of the same eye captured 3 weeks after the peeling showing regularization of the anterior curvature map with reduction of astigmatism of about 4 cylinders. Also, there was reduction in the corneal thickness on pachymetry map.

A 34-year-old- male patient, is a known case of advanced keratoconus in the left eye, for which he underwent deep anterior lamellar keratoplasty (LK) 2 years ago. The patient had quiet post-operative course and all sutures were removed 8 months after the surgery with BCVA of 2030 but eventually his vision started to decrease to a BCVA of 20400 at presentation. Upon examination, he was found to have quiet conjunctiva. Examination of the cornea showed suture-less corneal graft with sub-epithelial non-vascularized elevated corneal opacity that was more prominent in the temporal aspect of the graft measuring about 2.5 × 3 mm with faint smooth borders. The nasal aspect of the opacity was less dense compared to the temporal one with sparing of the visual axis. Otherwise, the graft was relatively clear with no evidence of underlying edema (Fig. 3a).

Topographically, there was an irregular corneal surface on the sagittal curvature map due to massive flattening effect caused by the corneal opacity temporally with total anterior astigmatism of 16 diopters, also there is evident anterior elevation corresponding to the area of opacity. on pachymetry map there was marked thickening at the area of the opacity temporally (Fig. 3b).

The patient was counseled regarding the surgical option for visual rehabilitation of his condition and he was offered simple peeling in the minor operating room under topical anesthesia, which was done in a similar fashion as in case 1 with no complications. The excised tissue was identical to the other 2 cases with thick irregular corneal epithelium, absent Bowman’s layer and sub-epithelial fibrosis but there was a small focal area of degenerative non-inflammatory pannus (Fig. 3c and d). Three weeks after the procedure the BCVA improved to 20100 with more clear graft (Fig. 3e). On topography, there was pronounced regularization of the anterior curvature map with reduction of astigmatism of about 4 cylinders due to mechanical removal of this sub-epitheliual opacity. Also, there was reduction in the overall corneal thickness on pachymetry map (Fig. 3f).

## 4 Discussion

Acquired Corneal Sub-epithelial Hypertrophy (ACSH) is a distinct histopathological corneal entity which can affect the vision due to irregularity in the corneal surface causing high irregular astigmatism and characteristic topographic findings in the form of an increased thickness and front elevation with normal back elevation [4]. Although this newly described condition shares the same histopathological findings as peripheral hypertrophic sub-epithelial corneal degeneration (PHSCD), they differ in terms of age at presentation, location, etiology, morphology, and progression of the condition [4,[6], [7], [8]]. ACSH has tendency to occur in male patients and it is usually an acquired condition following corneal surgery e.g. corneal transplantation as in our series and it is usually unilateral to the operated eye [4]. Al-Rajhi, who first proposed this entity, described ACSH in 10 eyes following corneal procedure, 7 out of which had (one or repeated) PKP procedure indicating that it was the most common surgical procedure resulting in such opacification. ACSH has tendency to occur in male patients and it is usually an acquired condition following corneal surgery e.g. LK or PK as in our 3 male cases and it is usually unilateral to the operated eye [4]. To the contrary, PHSCD is an idiopathic bilateral condition and more seen in females [3,8]. It has been recently recognized and is often compared to Salzmann’s nodular degeneration (SND), which is also mostly bilateral in up to 80% of the cases and is seen more often in women [9]. SND is highly related to ocular surface diseases such as trachoma, vernal disease, and phlyectnular keratitis as well as in association with uveitis and/or band keratopathy [10]. Table1 summarizes the differentiating features of ACSH from PHSCD and SND. The latter 2 conditions share similar histopathological and demographic findings that are consistent with a non-inflammatory degenerative process [11]. On the other hand, ACSH, which is a newly emerging entity is thought to be acquired and secondary to surgical intervention or chronic corneal disease thus it is mostly unilateral (unless the patient has had an intervention or disease involving both eyes). Our reported 3 cases clearly fit into ACSH rather than the above 2 conditions.

**Table 1:** Demographic and clinical characteristics of 3 entities that can be considered in the differential diagnosis of cases with superficial corneal opacifications.

Clinical entity	ACSH [4]	PHSCD	SND
*Gender*	More in males	More in females	More in females
*Laterality*	Mostly unilateral	Bilateral	Mostly bilateral Can be unilateral
*Etiology (presumed)*	Secondary Reactive to surgery or chronic eye disease	Primary idiopathic	Associated with ocular surface disease
*Location*	Mostly peripheral Can be central	Inter-palpebral Peripheral/nasal quadrant	Limbal Mid-peripheral
*Opacity appearance*	Arcuate, sectorial, annular, or diffuse Single	Variable	Small Nodular Multiple
*Management*	Peeling SK (rarely needed)	Peeling SK (rarely needed)	SK

**PHSCD**: Peripheral hypertrophic sub-epithelial corneal degeneration, **ACSH**: Acquired Corneal Sub-epithelial Hypertrophy, **SND**: Salzmann’s nodular degeneration, **SK:** Superficial keratectomy.

As in any very superficial corneal lesion in symptomatic patient and when medical therapy alone is likely to fail, membranectomy by simple peeling may be sufficient (because bowman’s layer is not deeply involved) with or without superficial keratectomy (the latter is rarely needed as a smooth surgical plane can be easily identified during peeling in cases of ASCH and PHSD as opposite to Salzmann’s nodular degermation (SND), in which finding this plane is more difficult due to deeper involvement) [2,6,7]. Mitomycin-c can be applied at the end of the procedure to reduce the risk of recurrence as this method was described for the excision of SND [2,7,12].

In our cases, superficial peeling was sufficient to achieve corneal clarity and improve vision.

The advantage of this treatment is that it is a minor surgical intervention with a faster recovery. Recurrence was not noted for 3 months in both of our patients, but long-term follow up is needed to detect any recurrence in these patients even though recurrence of opacity is not expected. The major limitation of this case series was the rarity of this condition, thus the limited number of patients included. Also, the lack of definite explanation and exact pathogenesis for the development of this condition. This warrants further multicentric collaborative study in the future to collect enough number of similar cases.

## 5 Conclusions

We believe that ophthalmologists should be aware of this newly described entity in order to be able to identify cases of ACSH and treat them accordingly with simple peeling or superficial keratectomy saving them from the more invasive penetrating keratoplasty procedure. Further ultrastructural and biochemical studies are needed to clarify the etiology, pathogenesis, and long-term outcome behind development of this specific type of corneal opacity especially following LK or PKP for possible preventive measures.

## Funding

The Case series has been supported by King Saud University Medical City, however there were no funds involved.

## Ethical approval

This case report was prepared in accordance with the ethical standards of the human ethics in accordance with the Helsinki Declaration. Case reports do not require IRB approval; however, the General Informed Consent includes patients’ approval for use of relevant clinical and surgical information in an anonymous way.

## Consent

General informed consent has been taken from all patients for use of relevant clinical and surgical information in an anonymous way.

## Author contribution

First and second authors: Collection of data, literature review, and drafting the manuscript.

Third, fourth and fifth authors: Clinical diagnosis and surgical management of the patients.

Last senior author: Critical review of the manuscript, histopathological diagnosis, and corresponding author.

## Registration of research studies

Registration has been obtained: #esearchregistry5315.

## Guarantor

Dr. Hind Alkatan.

## Provenance and peer review

Not commissioned, externally peer-reviewed.

## Declaration of Competing Interest

The authors declare that they have no competing or conflict of interest, and the General Informed Consent includes patients’ approval for use of relevant clinical and surgical information in an anonymous way.

## References

[1] Järventausta P.J., Tervo T.M., Kivelä T., Holopainen J.M. (2014). Peripheral hypertrophic subepithelial corneal degeneration – clinical and histopathological features. Acta Ophthalmol..

[2] Gore D.M., Iovieno A., Connell B.J., Alexander R., Meligonis G., Dart J.K. (2013). Peripheral hypertrophic subepithelial corneal degeneration: nomenclature, phenotypes, and long-term outcomes. Ophthalmology.

[3] Rommel F., Grisanti S., Ranjbar M. (2017). Peripheral hypertrophic subepithelial corneal degeneration. JAMA Ophthalmol..

[4] Al-Rajhi A.A., Helmi H.A., Alkatan H.M., Al-Obailan M., Al-Rajhi A. (2019). Successful treatment of corneal opacification with associated thickened epithelium by simple peeling: acquired corneal subepithelial hypertrophy (ACSH). Saudi J. Ophthalmol..

[5] Agha R.A., Borrelli M.R., Farwana R., Koshy K., Fowler A., Orgill D.P., SCARE Group (2018). The PROCESS 2018 statement: updating consensus Preferred Reporting of CasE Series in Surgery (PROCESS) guidelines. Int. J. Surg..

[6] Wood T.O., Griffith M.E. (1988). Surgery for corneal epithelial basement membrane dystrophy. Ophthalmic Surg..

[7] Bowers P.J., Price M.O., Zeldes S.S., Price F.W. (2003). Superficial keratectomy with mitomycin-C for the treatment of Salzmann’s nodules. J. Cataract Refract. Surg..

[8] Schargus M., Kusserow C., Schlötzer-Schrehardt U., Hofmann-Rummelt G., Schlunck G., Geerling G. (2015). Peripheral hypertrophic subepithelial corneal degeneration presenting with bilateral nasal and temporal. Eye.

[9] Jaworski A., Arvanitis A. (1999). Salzmann’s nodular degeneration of the cornea. Clin. Exp. Optom..

[10] Hirst L.W., Farmer E.R., Green W.R., Silver A., Walsh F.B. (1984). Familial corneal scarring: a new dystrophy?. Ophthalmology.

[11] Morais L.T.M., Basile Neto A., Carvalho L.S., Silva Neto J.R., Lima M.H.C. (2019). Peripheral hypertrophic subepithelial corneal degeneration: phenotypical description. eOftalmo.

[12] Khaireddin R., Katz T., Baile R.B., Richard G., Linke S.J. (2011). Superficial keratectomy, PTK, and mitomycin C as a combined treatment option for Salzmann’s nodular degeneration: a follow-up of eight eyes. Graefes Arch. Clin. Exp. Ophthalmol..

